# Statistical Perspective on Functional and Causal Neural Connectomics: A Comparative Study

**DOI:** 10.3389/fnsys.2022.817962

**Published:** 2022-03-02

**Authors:** Rahul Biswas, Eli Shlizerman

**Affiliations:** ^1^Department of Statistics, University of Washington, Seattle, WA, United States; ^2^Department of Applied Mathematics, Department of Electrical & Computer Engineering, University of Washington, Seattle, WA, United States

**Keywords:** neural connectivity, connectome, mapping network, functional connectivity, probabilistic graphical models, causal connectivity

## Abstract

Representation of brain network interactions is fundamental to the translation of neural structure to brain function. As such, methodologies for mapping neural interactions into structural models, i.e., inference of functional connectome from neural recordings, are key for the study of brain networks. While multiple approaches have been proposed for functional connectomics based on statistical associations between neural activity, association does not necessarily incorporate causation. Additional approaches have been proposed to incorporate aspects of causality to turn functional connectomes into causal functional connectomes, however, these methodologies typically focus on specific aspects of causality. This warrants a systematic statistical framework for causal functional connectomics that defines the foundations of common aspects of causality. Such a framework can assist in contrasting existing approaches and to guide development of further causal methodologies. In this work, we develop such a statistical guide. In particular, we consolidate the notions of associations and representations of neural interaction, i.e., types of neural connectomics, and then describe causal modeling in the statistics literature. We particularly focus on the introduction of directed Markov graphical models as a framework through which we define the Directed Markov Property—an essential criterion for examining the causality of proposed functional connectomes. We demonstrate how based on these notions, a comparative study of several existing approaches for finding causal functional connectivity from neural activity can be conducted. We proceed by providing an outlook ahead regarding the additional properties that future approaches could include to thoroughly address causality.

## 1. Introduction

The term “connectome” typically refers to a network of neurons and their anatomical links, such as chemical and electrical synapses. The connectome represents the anatomical map of the neural circuitry of the brain (Sporns et al., 2005). Connectome mapping can be achieved with the help of imaging techniques and computer vision methods at different scales (Shi and Toga, 2017; Sarwar et al., 2020; Xu et al., 2020). The aim of finding the connectome is to provide insight into how neurons are connected and how they interact to form brain function.

While the anatomical connectome includes the backbone information on possible ways of how neurons could interact, it does not fully reflect the “wiring diagram of the brain”, which is expected to incorporate the dynamic nature of neurons' activity and their interactions (Lee and Reid, 2011; Kopell et al., 2014; Kim et al., 2019a,b). In particular, the anatomical connectome does not correspond to how the anatomical structure relates to brain function since each anatomical connectivity map can encode several functional outcomes of the brain (Bargmann and Marder, 2013). Thereby, the term connectome has been extended beyond the anatomical meaning. In particular, a map that reflects neurons functions is named Functional Connectome (FC) and it represents the network of associations between neurons with respect to their activity over time (Reid, 2012). Finding FC is expected to lead to more fundamental understanding of brain function and dysfunction (Hassabis et al., 2017). Indeed, FC is expected to include and facilitate inference of the governing neuronal pathways essential for brain functioning and behavior (Finn et al., 2015). Two neurons are said to be functionally connected if there is a significant relationship between their activity over time where the activity can be recorded from neurons over time and measured with various measures (Shlizerman et al., 2012). In contrast to the anatomical connectome, the functional connectome needs to be inferred, as it cannot be directly observed or mapped, since the transformation from activity to associations is intricate.

Several approaches have been introduced to infer the FC. These include approaches based on measuring correlations, such as pairwise correlation (Rogers et al., 2007; Preti et al., 2017), or sparse covariance matrix that is comparatively better than correlations given limited time points (Xu and Lindquist, 2015; Wee et al., 2016). Furthermore, for such scenarios, regularized precision matrix approaches were proposed to better incorporate conditional dependencies between neural time courses, where the precision matrix is inferred by a penalized maximum likelihood to promote sparsity (Friedman et al., 2008; Varoquaux et al., 2010; Smith et al., 2011). While there is a wide variety of methods, there is still a lack of unification as to what defines the “functional connectome.” A taxonomy which provides a systematic treatment grounded from definitions and followed with algorithmic properties is currently unavailable. An existing taxonomy for FC considers generic aspects, such as, undirected and directed, model-based and model-free, time and frequency-domains (Bastos and Schoffelen, 2016). Here, we consider the angle of association and causation, such as pairwise association vs. non-pairwise graphical associations.

Moreover, the prevalent research on FC, outlined above, deals with finding associations between neural signals in a non-causal manner. That is, in such a mapping we would know that a neuron A and a neuron B are active in a correlated manner, however, we would not know whether the activity in neuron *A* causes neuron *B* to be active (*A*→*B*), or is it the other way around (*B*→*A*)? Or, is there a neuron *C* which intermediates the correlation between *A* and *B* (*A*←*C*→*B*)? In short, questions of this nature distinguish causation from association.

In this work, we provide a statistical framework for FC and investigate the aspect of causality in the notion of Causal Functional Connectome (CFC) which would answer the aforementioned causal questions (Ramsey et al., 2010; Valdes-Sosa et al., 2011). Specifically, we introduce the directed Markov graphical models as a framework for the representation of functional connectome and define the Directed Markov Property—an essential criterion for examining the causality of proposed functional connectomes. The framework that we introduce allows us to delineate the following properties for a statistical description of causal modeling

1. Format of causality.
2. Inclusion of temporal relationships in the model.
3. Generalization of the statistical model.
4. Dependence on parametric equations.
5. Estimation-based vs. hypothesis test-based inference of CFC from recorded data.
6. Inclusion of cycles and self-loops.
7. Incorporation of intervention queries in a counterfactual manner.
8. Ability to recover relationships between neurons when ground-truth dynamic equation of neural activity are given.

We discuss the applicability and the challenges of existing approaches for CFC inference with respect to these statistical properties. In particular, we compare existing approaches for causal functional connectome inference, such as Granger Causality (GC), Dynamic Causal Modeling (DCM) and Directed Probabilistic Graphical Models (DPGM) based on these properties. The comparative study provides a taxonomy of causal properties that existing methods address and an outlook of the properties for future extensions to address.

The following is a list of acronyms used in this paper: Functional Connectivity (FC), Causal Functional Connectivity (CFC), Granger Causality (GC), Dynamic Causal Model (DCM), Directed Probabilistic Graphical Model (DPGM), Directed Markov Property (DMP), Functional Magnetic Resonance Imaging (fMRI), Diffusion Tensor Imaging (DTI), Electroencephalography (EEG), Magnetoencephalography (MEG), Associative Functional Connectivity (AFC), Probabilistic Graphical Model (PGM), Markov Property (MP), Lateral geniculate nucleus (LGN), Visual cortex (VC), Superior colliculus (SC), Pulvinar (P), Central nucleus of the amygdala (CeA), paraventricular nucleus (PVN), hypothalamus-pituitary-adrenal axis (HPA), Directed Acyclic Graph (DAG), Peter Clark (PC), Greedy Equivalence Search (GES), Greedy Interventional Equivalence Search (GIES), Continuous Time Recurrent Neural Network (CTRNN), Accuracy (A), Sensitivity (S), True Positive (TP), False Positive (FP), True Negative (TN), False Negative (FN).

## 2. Neural Connectomics: Anatomical and Functional

Recent advances in neuro-imaging has made it possible to examine brain connectivity at micro and macro scales. These, for example, include electron microscopy reconstruction of the full nervous system with neurons and synapses of *C. elegans* in the mid-1980s (White et al., 1986). Recent non-invasive diffusion tensor imaging, followed by computational tractography, allow to recover fiber tract pathways in human brain (Conturo et al., 1999; Catani et al., 2003; Le Bihan, 2003). Also, two-photon tomography facilitates imaging axonal projections in the brain of mice (Ragan et al., 2012). Although the anatomical reconstruction gives insights into the building blocks and wiring of the brain, how that leads to function remains unresolved. A representative example is *C. elegans*, in which connections and neurons were fairly rapidly mapped and some neurons were associated with functions, it is still unclear what most of the connections “do” (Morone and Makse, 2019).

It became increasingly clear that anatomical wiring diagram of the brain could generate hypotheses for testing, but it is far from the resolution of how the anatomical structure relates to function and behavior. This is because each wiring diagram can encode several functional and behavioral outcomes (Bargmann and Marder, 2013). In both vertebrate and invertebrate brains, pairs of neurons can influence each other through several parallel pathways consisting of chemical and electrical synapses, where the different pathways can either result in similar or dissimilar behavior. Furthermore, neuromodulators re-configure fast chemical and electrical synapses and have been shown to act as key mediators for various brain states. Given this background, it is generally agreed upon that the anatomical synaptic connectivity does not provide adequate information to predict the physiological output of neural circuits. To understand the latter, one needs to understand the flow of information in the network, and for that, there is no substitute for recording neuronal activity and inferring functional associations from it. This is what the functional connectome aims to achieve.

The functional connectome is the network of information flow between neurons based on their activity and incorporates the dynamic nature of neuronal activity and interactions between them. To obtain neuronal activity and dynamics, the neuronal circuit needs to be monitored and/or manipulated. Recent approaches to record such activity include brain wide two-photon calcium single neuron imaging *in vivo* of *C. elegans* (Kato et al., 2015), wide-field calcium imaging of regions of the brain of behaving mice (Zatka-Haas et al., 2020), two-photon calcium imaging (Villette et al., 2019), Neuropixel recordings of single neurons in the brain of behaving mice (Steinmetz et al., 2018, 2019), and functional Magnetic Resonance Imaging (fMRI) recordings of voxels in the human brain as part of the Human Connectome Project (Van Essen et al., 2012; Stocco et al., 2019). It is noteworthy that FC aims to capture statistical dependencies based on neural recordings and does not rely on the underlying anatomical connectivity, thereby FC methods are applicable for different scales of neural data—micro (Hill et al., 2012), meso (Passamonti et al., 2015), and macro (Mumford and Ramsey, 2014). Yet, when the underlying anatomical connectivity is charted, e.g., macro-scale anatomical connectivity in Diffusion Tensor Imaging (Li et al., 2013), it can be used to further constrain the inference of FC (Bowman et al., 2012).

In this section, we set the notions of the neural connectome with regards to anatomical and functional aspects. These notions are from a statistical perspective and will assist to connect connectomics in further sections with causation.

### 2.1. Anatomical Connectome

Let us consider a brain network *V* = {*v*_1_, …, *v*_*N*_} with *N* neurons labeled as *v*_1_, …, *v*_*N*_. We will denote the edges as *E*_*a*_⊂*V* × *V* between pairs of neurons that correspond to anatomical connectivity between the neurons (Sporns et al., 2005). We will refer to the graph *G* = (*V, E*_*a*_) as the anatomical connectome between the neurons in *V*. Each edge (*v, u*) ∈ *E*_*a*_ will be marked by a weight *w*_*vu*_ ∈ ℝ that quantifies the strength of the anatomical connection from *v* to *u*.

#### 2.1.1. Examples

##### 2.1.1.1. Binary Gap Connectome

If there is a gap junction connection from neuron *v* to neuron *u*, the set *E*_*a*_ will include the edges *v*→*u* and *u*→*v* and they will be marked with weight 1 (Jarrell et al., 2012). The resulting graph is undirected in the sense that (*v, u*) ∈ *E*_*a*_ iff (*u, v*) ∈ *E*_*a*_. The resulting weight matrix with entries *w*_*vu*_ is symmetric.

##### 2.1.1.2. Weighted Synaptic Connectome

If there is a synaptic connection from neuron *v* to neuron *u*, the set *E*_*a*_ will include the edge *v*→*u* and it will be marked with a weight which is equal to the number of synaptic connections starting from neuron *v* to neuron *u*. The resulting graph is a directed graph in the sense that (*v, u*) ∈ *E*_*a*_ does not imply (*u, v*) ∈ *E*_*a*_, and the weight matrix (*w*_*vu*_) is asymmetric (Varshney et al., 2011).

### 2.2. Functional Connectome (Associative)

*Functional Connectome* (FC) is a graph with nodes being the neurons in *V* and pairwise edges representing a stochastic relationship between the activity of the neurons. Weights of the edges describe the strength of the relationship. Let *X*_*v*_(*t*) ∈ ℝ denote a random variable measuring the activity of neuron *v* at time *t*. Examples for such variables are instantaneous membrane potential, instantaneous firing rate, etc. *Associative FC* (AFC) is an undirected graph, *G* = (*V, E*), where *E*_*v,u*_ ∈ *E*, an undirected edge between *v* and *u*, represents stochastic association between neuron *v* and *u*. Edge weights signify the strength of the associations. Different approaches define stochastic associations leading to different candidates for AFC, as follows.

#### 2.2.1. Pairwise Associative Connectivity

We first describe pairwise stochastic associations (*pairwise AFC*). Let us consider recordings at time points 0, …, *T*, with activities *X*_*v*_ = {*X*_*v*_(*t*):*t* ∈ 0, …, *T*} for neuron *v*. The following measures of pairwise association will correspond to pairwise AFC.

##### 2.2.1.1. Pearson's Correlation

The Pearson's correlation between *X*_*v*_ and *X*_*u*_ is defined as


$$r\left(X_{v},X_{u}\right)=\frac{\sum_{t=0}^{T}\left(X_{v}\left(t\right)-{\bar{X}}_{v}\right)\left(X_{u}\left(t\right)-{\bar{X}}_{u}\right)}{\sqrt{\sum_{t=0}^{T}{\left(X_{v}\left(t\right)-{\bar{X}}_{v}\right)}^{2}\sum_{t=0}^{T}{\left(X_{u}\left(t\right)-{\bar{X}}_{u}\right)}^{2}}}$$

where ${\bar{X}}_{v}=\frac{1}{T+1}\sum_{t=0}^{T}X_{v}\left(t\right)$, for *v* ∈ *V*. Pearson's correlation takes a value between –1 and 1 and the further its value is from 0, the larger is the degree of pairwise association. Neurons *v* and *u* are connected by pairwise AFC with respect to Pearson's correlation if *r*(*X*_*v*_, *X*_*u*_) is greater than a threshold in absolute value and the value *r*(*X*_*v*_, *X*_*u*_) would be the weight of the connection, i.e., *E*_*v,u*_ = thresh(*r*(*X*_*v*_, *X*_*u*_)). Correlation coefficient and the AFC based on it are sensitive to indirect third party effects such as an intermediary neuron, poly-synaptic influences, indirect influences, and noise.

##### 2.2.1.2. Partial Correlation

Partial correlation is an additional measure of pairwise stochastic association between random variables defined as follows. Let the covariance between *X*_*v*_ and *X*_*u*_ be $C_{vu}=\frac{1}{T+1}\sum_{t=0}^{T}\left(X_{v}\left(t\right)-{\bar{X}}_{v}\right)\left(X_{u}\left(t\right)-{\bar{X}}_{u}\right)$. The matrix of covariances, Σ = (_*C*_*vu*_)1 ≤ *v, u* ≤ *N*_ is called the covariance matrix for the variables *X*_1_, …, *X*_*N*_. Let the *v, u*-th entry of the inverse covariance matrix Σ^−1^ be γ_*vu*_. The partial correlation between *X*_*v*_ and *X*_*u*_ is defined as


$$\rho \left(X_{v},X_{u}\right)=-\frac{\gamma_{vu}}{\sqrt{\gamma_{vv}\gamma_{uu}}}$$

Partial correlation rectifies the problem of correlation coefficient being sensitive to third party effects since it estimates the correlation between two nodes after removing the variance shared with other system elements. Partial correlation takes a value between –1 and 1 and the further its value is from 0, the larger is the degree of pairwise association. Neurons *v* and *u* are connected by pairwise AFC with respect to partial correlation, if ρ(*X*_*v*_, *X*_*u*_) is greater than a threshold in absolute value and the value ρ(*X*_*v*_, *X*_*u*_) would be the weight of the connection, i.e., *E*_*v,u*_ = thresh(ρ(*X*_*v*_, *X*_*u*_)).

#### 2.2.2. Undirected Probabilistic Graphical Model

**Figure 1:**
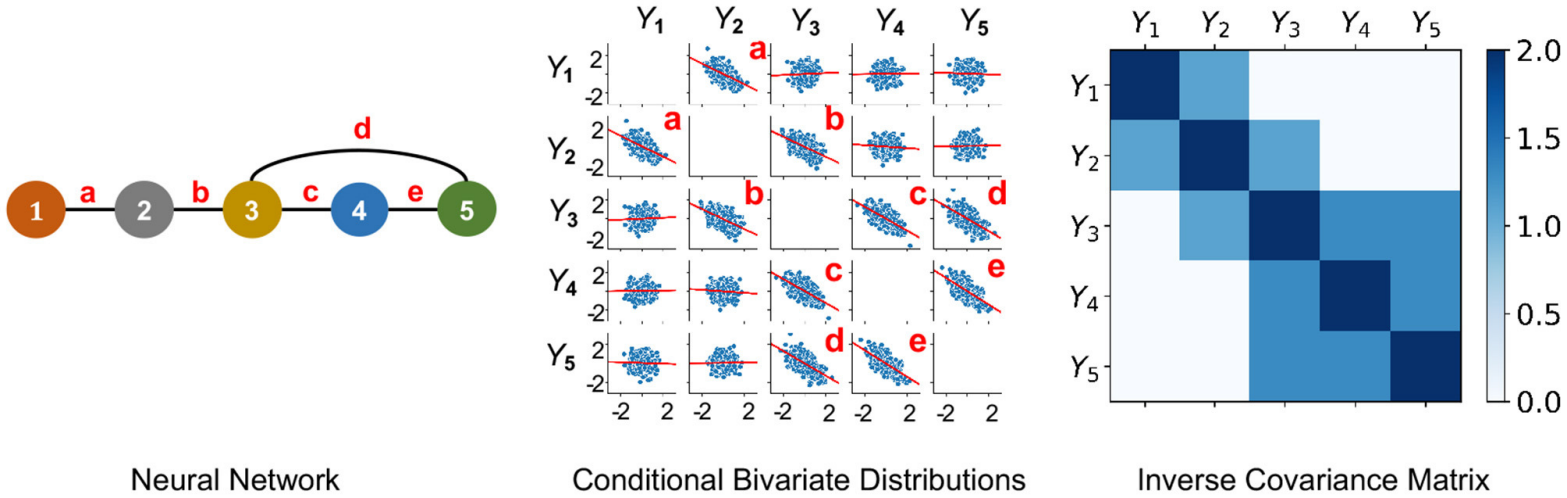
An undirected PGM and its Markov Property. Left: Network of 5 neurons that is defined in Example 2.1. Neurons are labeled as 1−5 and edges are labeled as a-e. Middle: *Y*_1_, …, *Y*_5_ following centered multivariate Gaussian distribution with entries of inverse covariance matrix γ_*ij*_ such that γ_13_ = γ_14_ = γ_15_ = γ_24_ = γ_25_ = 0, when it factorizes with respect to the Neural Network. This plot shows conditional bivariate distributions given other variables ∈ (−0.2, 0.2) and demonstrates (Equation 1) where *Y*_*v*_ and *Y*_*u*_ are not correlated conditional on other variables (seen by nearly flat red trend-lines) for (*v, u*) not an edge of the Neural Network. This indicates that *Y*_1_, …, *Y*_5_ satisfies the Markov Property with the Neural Network. Right: Due to a Gaussian distribution, the non-zero entries of the Inverse Covariance Matrix of *Y*_1_, …, *Y*_5_ correspond to the edges of the Neural Network.

Undirected Probabilistic Graphical Models (PGM) allow for modeling and infering stochastic associations while considering multi-nodal interactions beyond pairwise manner through a graphical model. Let *G* = (*V, E*) be an undirected graph with neuron labels *V* = {*v*_1_, …, *v*_*N*_} and edges *E* (Figure 1). Let *Y*_*v*_ denote a scalar-valued random variable corresponding to *v* ∈ *V*, for example, *Y*_*v*_ can be the value of *X*_*v*_ at recording time *t*: *Y*_*v*_ = *X*_*v*_(*t*), or average of recordings over time: $Y_{v}={\bar{X}}_{v}$, etc. For a set of neurons *A*⊂*V*, let *Y*_*A*_ denote the random vector (*Y*_*v*_, *v* ∈ *A*). The random vector *Y*_*V*_, is said to satisfy the *undirected graphical model* with graph *G*, if, *Y*_*v*_ is conditionally independent of *Y*_*u*_ given *Y*_*V*\{*v, u*}_ for (*v, u*)∉*E*, denoted as


(1) $$\begin{array}{r} Y_{v}⊥⊥Y_{u}|Y_{V\backslash \left\{v,u\right\}}\text{for}\left(v,u\right)\notin E. \end{array}$$

When (Equation 1) holds, *Y*_*v*_ is said to satisfy the *Markov property* with the undirected graph *G* (Wainwright and Jordan, 2008). The Markov property with undirected graph *G* translates each *absent* edge between a pair of nodes *v* and *u* into *conditional independence* of *Y*_*v*_ and *Y*_*u*_ given all other nodes. In other words, nodes in the undirected PGM that are not connected by an edge appear as non-interacting when all the nodes are examined.

** Definition 1**. The *AFC* for neurons in *V* is the undirected graph *G* = (*V, E*) such that (*Y*_*v*_, *v* ∈ *V*) satisfies the *Markov Property* with respect to *G*. When *Y*_*v*_ = *X*_*v*_(*t*), the associative FC is contemporaneous at each time *t*.

According to this definition, the graph edges *E*_*v,u*_ are well-defined. With respect to their weights, there are no unique candidates. When (*Y*_*v*_, *v* ∈ *V*) follows a multivariate Gaussian distribution, an edge is present if and only if the partial correlation between them is non-zero. Thereby, in practice, partial correlation ρ(*X*_*v*_, *X*_*u*_) is typically used for weight of edge *E*_*v,u*_ ∈ *E* (Figure 1).

In the above we have defined AFC to be the undirected graphical model that has the Markov Property. A natural question that arises is what kind of probability distributions for the graphical model allow it to follow the Markov Property? The Factorization Theorem by Hammersley, Clifford and Besag prescribes conditions on the probability distributions of *Y*_*v*_, *v* ∈ *V* for which undirected graphical model has the Markov property (Drton and Maathuis, 2017).

** Theorem 1 (Factorization Theorem)**. If *Y*_*v*_, *v* ∈ *V* has a positive and continuous density *f* with respect to the Lebesgue measure or is discrete with positive joint probabilities, then it satisfies the Markov property (Equation 1) with respect to *G* = (*V, E*) if and only if the distribution of *Y*_*v*_, *v* ∈ *V* *factorizes* with respect to *G*, which means,


(2) $$\begin{array}{r} f\left(y\right)=\underset{C\subset G:C\text{is complete}}{\prod }\phi_{C}\left(y_{C}\right),y\in {\mathbb{R}}^{V} \end{array}$$

where, *f* is the density of *Y*_*v*_, *v* ∈ *V*, ϕ_*C*_ is an arbitrary function with domain ℝ^*C*^, and *y*_*C*_ is the sub-vector (*y*_*v*_:*v* ∈ *C*) and *C*⊂*G* is complete, i.e., *E*_*v,u*_ ∈ *E* for all *v*≠*u* ∈ *C*, are connected by an edge.

Under the multivariate Gaussian assumption, Theorem 1 yields a simple prescription for obtaining the undirected graph with the Markov Property. When *Y*_*v*_, *v* ∈ *V* are distributed as multivariate Gaussian with positive definite covariance matrix Σ, then *G* is determined by the zeroes of the inverse covariance matrix Σ^−1^, i.e., *E*_*v, w*_ ∈ *E* iff $\Sigma_{ij}^{-1}\neq 0$. This is illustrated in Example 2.1 and Figure 1. This has been used for inferring undirected PGMs in several applications (Epskamp et al., 2018; Dyrba et al., 2020). In such a case, estimation of *G* is tantamount to estimation of Σ^−1^. Methods for estimation of Σ^−1^ include Maximum Likelihood Estimation (MLE) which provides non-sparse estimates of Σ^−1^ (Speed and Kiiveri, 1986) and penalized MLE, e.g., by Graphical Lasso, which provides sparse estimates of Σ^−1^, with the estimates being statistically consistent under assumptions (Meinshausen and Bühlmann, 2006; Banerjee et al., 2008; Rothman et al., 2008; Shojaie and Michailidis, 2010).

**Example 2.1** (Markov Property and Multivariate Gaussian Assumption). Let us consider an example with 5 neurons. Suppose (*X*_1_(*t*), …, *X*_5_(*t*)) follow a centered multivariate Gaussian distribution with positive definite covariance matrix Σ and independent copies over time *t*. Let $\Sigma^{-1}={\left(\gamma_{ij}\right)}_{1\leq i,j\leq N}$ be the inverse covariance matrix. The probability density of (*X*_1_(*t*), …, *X*_5_(*t*)) is given by


(3) $$\begin{array}{r} f\left(x^{t}\right)\propto \exp\left(-\frac{1}{2}\overset{5}{\underset{i,j=1}{\sum }}\gamma_{ij}x_{i}^{t}x_{j}^{t}\right),x^{t}\in {\mathbb{R}}^{5}. \end{array}$$

The neural network graph is illustrated in Figure 1-left and we note that the graph has the following complete subsets


{1,2},{2,3},{3,4},{4,5},{3,5},{3,4,5}.

Thereby, for each *t*, it is observed that γ_13_ = γ_14_ = γ_15_ = γ_24_ = γ_25_ = 0 if and only if the density in Equation (3) factorizes as


$$\begin{array}{l} f\left(x^{t}\right)\propto \exp\left(-\frac{1}{2}\left(\gamma_{11}{x_{1}^{t}}^{2}+\gamma_{12}x_{1}^{t}x_{2}^{t}+\gamma_{22}{x_{2}^{t}}^{2}\right)\right)\exp\left(-\frac{1}{2}\gamma_{23}x_{2}^{t}x_{3}^{t}\right)\\ \;\exp\left(-\frac{1}{2}\left(\gamma_{33}{x_{3}^{t}}^{2}+\gamma_{34}x_{3}^{t}x_{4}^{t}+\gamma_{4}{x_{4}^{t}}^{2}\right)\right)\\ \;\exp\left(-\frac{1}{2}\left(\gamma_{45}x_{4}^{t}x_{5}^{t}+\gamma_{55}{x_{5}^{t}}^{2}\right)\right)\exp\left(-\frac{1}{2}\gamma_{35}x_{3}^{t}x_{5}^{t}\right) \end{array}$$

That is, the density in Equation (3) factorizes according to Equation (2) with respect to the graph. Hence, when γ_13_ = γ_14_ = γ_15_ = γ_24_ = γ_25_ = 0, according to Theorem 1 it follows that (*X*_1_(*t*), …, *X*_5_(*t*)) satisfies the Markov Property with respect to the graph in Figure 1-left.

## 3. From Association to Causation

**Figure 2:**
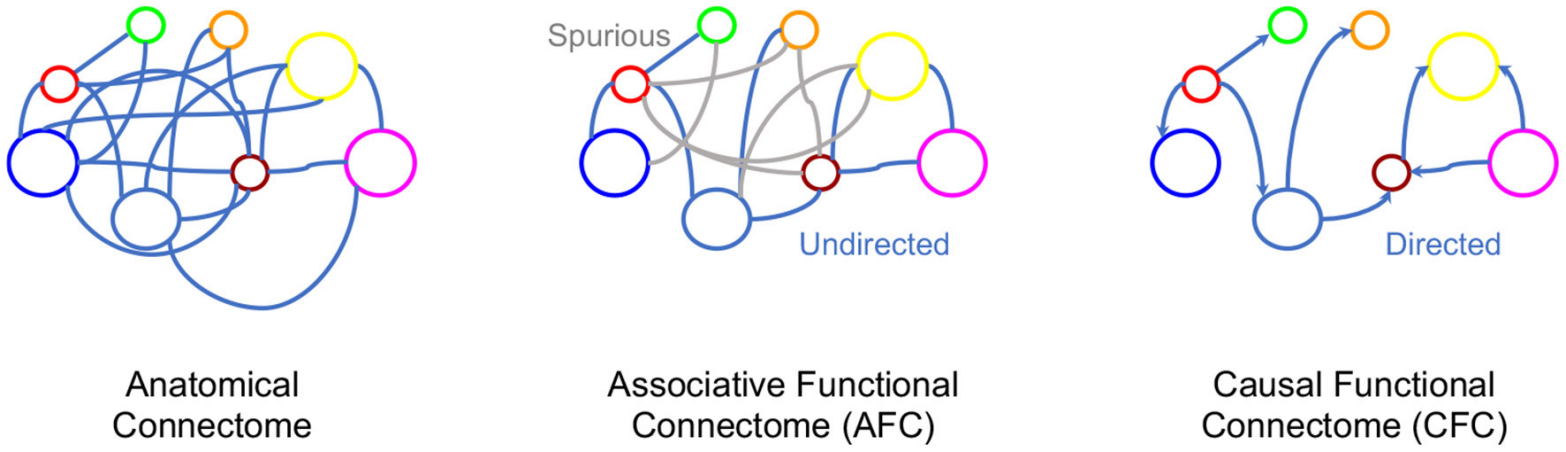
Various connectome representations for the same network of neurons. Left to right: Anatomical connectivity between brain regions, an undirected graph representing AFC where gray indicates spurious edges, and a directed graph with directions representing causal relationships.

In Section 2.2 we provided a systematic exposition of AFC, however, the ultimate phenomenon of functional connectivity is to capture the causal interaction between neural entities (Horwitz, 2003; Ramsey et al., 2010; Friston, 2011; Valdes-Sosa et al., 2011). Indeed, FC research is already targeting addition of causal statements to associative FC by identifying correlated brain regions as causal entities, suggesting the need to incorporate functional connectivity beyond association (Smith et al., 2009; Power et al., 2011; Yeo et al., 2011; Power and Petersen, 2013). From associations alone, causal inference is ambiguous with possible parallel, bidirectional and spurious causal pathways. Narrowing the space of causal pathways inferred from brain signals can significantly progress FC toward its aims of finding causal neural interactions (see Figure 2). For example, in a neural circuit, an edge between neuron A and neuron B could mean either neuron A influences neuron B or vice versa. This directionality of influence is unclear from the association. In the instance when neuron C influences activity of both A and B neurons, while they do not influence each other, a spurious association would be found between A and B in the AFC (see example in Figure 2). Recent progress in causal inference in statistics literature has made it possible to define causality and make causal inferences. Given this background, we review causal modeling in statistics to aid the development of a novel framework for *Causal Functional Connectivity* (CFC) from neural dynamics.

### 3.1. Causal Modeling

**Figure 3:**
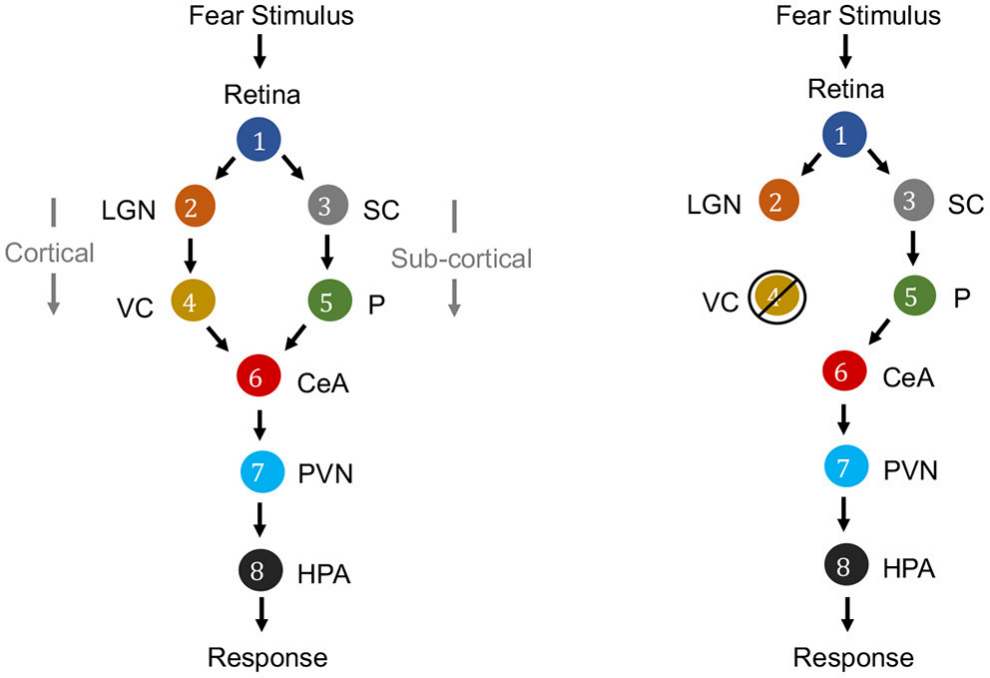
Causal graphs for electrical activity of brain nuclei for fear perception from visual stimulus. Left: Literature describes two routes for fear stimulus propagation from Retina to HPA: Retina → LGN → VC → CeA → PVN → HPA (cortical route) and Retina → SC → P → CeA → PVN → HPA (subcortical route). Right: It is demonstrated that even if there is intervention by ablation or lesion in the striate cortex of VC, thereby blindness, yet fear response to visual stimuli (“blindsight”) is yielded through the subcortical route (Morris et al., 1999; Carr, 2015).

Causation is a relation between two events, in which one event is the cause and the other is the effect. In the context of neuroscience, causality is a major factor. For example, in fear perception in the human brain, the causal relationships among the activity of retina, lateral geniculate nucleus (LGN), visual cortex (VC), superior colliculus (SC), pulvinar (P), central nucleus of the amygdala (CeA), paraventricular nucleus (PVN) and hypothalamus-pituitary-adrenal axis (HPA) can be considered (see Figure 3). While the information flows from the Retina to HPA, there is no direct link between them. Fear stimulus of the retina causes trigerring of the HPA (response region) mediated by the activity of intermediate brain nuclei in a specific sequence of activation through two merging pathways (Pessoa and Adolphs, 2010; Bertini et al., 2013; Carr, 2015).

A causal model relates the cause and effect, rather than recording correlation in the data and allows the investigator to answer a variety of queries such as *associational queries* (e.g., having observed activity in LGN, what activity can we expect in CeA?), *abductive queries* (e.g., what are highly plausible explanations for active CeA during fear stimulus?), and *interventional queries* (e.g., what will happen to the causal pathways if there is an ablation of VC?). Often interventional queries are especially of interest in neural connectomics, with interventions including neural ablation and external neuromodulation (Bargmann and Marder, 2013; Horn and Fox, 2020). In such cases, causal modeling aims to correctly predict the effect of an intervention in a counterfactual manner, that is, without necessarily performing the intervention but from observational data (Pearl, 2009a).

#### 3.1.1. Representing Causal Relations With a Directed Graph

Directed graphs provide convenient means for expressing causal relations among the variables. The vertices correspond to variables, and directed edges between the vertices represent a causal relationship that holds between pairs of variables. Formally, a graph consists of a set *V* of vertices (nodes) and a set *E*⊂*V* × *V* of edges that connect some pairs of vertices. Each edge can be either directed or undirected. In a directed graph, all edges are directed. A directed acyclic graph (DAG) is a directed graph without cycles. The directed graph representing causal relationships is called a causal graph. Figure 3 is a causal graph among eight variables representing electrical activity in eight brain regions. If G is a causal graph and there is a directed path from nodes *a* to *b* it implies that, the variable corresponding to node *a* is a cause for the variable corresponding to node *b*. For example, the electrical activity in Retina (node 1) is a common cause for electrical activity in LGN (node 2) and SC (node 3).

If there is any intervention of one of the variables such as ablation or neuromodulation of nodes, the network topology can be adapted with minor changes. In Figure 3 (right), to represent an ablation of visual cortex (VC), one would delete from the network all links incident to and originating from the node VC. To represent control of activity of VC by neuromodulation, one would delete all edges only incident to VC as then VC is not causally influenced by it's parent region's activity but by external neuromodulation.

#### 3.1.2. Statistical Properties of CFC Modeling

In the following we outline several statistical properties that are relevant in the context of causal modeling of functional connectivity.

1. *Format of causality*. This specifies how causation is defined in the model with respect to parameters or properties satisfied by the model.
2. *Inclusion of temporal relationships in the model*. Since the activity of neurons are related over time, this condition specifies whether such temporal relationships are incorporated in defining causal relationships among neural activity in the CFC model.
3. *Generalization of the statistical model*. This condition specifies model restrictions, such as linear or non-linear modeling, and informs whether such restrictions can be generalized.
4. *Parametric or non-parametric model*. This specifies whether the model is parametric (i.e., consisting of a parametric equation and needing estimation of parameters) or non-parametric (i.e., free of a parametric equation) (Casella and Berger, 2002). Non-parametric models have the advantage of not requiring assumptions on specific dynamical equations for the neural dynamics.
5. *Estimation-based vs. hypothesis test-based inference of CFC from recorded data*. Approaches for inferring CFC either support estimation of the CFC from the data or test of significance of hypothetical CFC models based on data. This condition specifies which category among these does the CFC model belong to.
6. *Inclusion of cycles and self-loops in the model*. Neural activity often consist of feedback loops and cycles (Byrne et al., 2014). This condition specifies whether such cycles and self-loops are represented in the CFC model.
7. *Incorporation of intervention queries in a counterfactual manner*. This condition specifies whether interventional queries are answered directly by the CFC model from observational data without performing the experimental intervention.
8. *Ability to recover relationships between neurons when ground-truth dynamic equation of neural activity are given*. It is often desirable that causal relationships between neural activity in ground truth dynamical equations are accurately represented by the inferred CFC (Schmidt et al., 2016; Reid et al., 2019). This condition sheds light into the performance of the CFC approach to recover such ground truth relationships from dynamical equations.

We proceed to delineate causation from statistical associations by surveying existing approaches, and describe relation of each approach to above statistical properties (Pearl, 2000, 2009a,b).

### 3.2. Granger Causality

*Granger causality* (GC) is a statistical methodology for determining whether one time series is useful in predicting another (Granger, 1969; Basu et al., 2015). Denoting *X*_*v*_(*t*) to be the state of neuron *v* at time *t*, the main idea of GC is that, *X*_*j*_ “Granger-causes” *X*_*i*_ if past of *X*_*j*_ contains information that helps predict the future of *X*_*i*_ better than using the information in the past of *X*_*i*_ or past of other conditioning variables *X*_*k*_ (Friston et al., 2013). There are several variations of Granger Causality (also called Wiener-Granger Causality), based on linear vector auto-regressive model (Lütkepohl, 2005), nonlinear vector auto-regressive model (Tank et al., 2018), and non-parametric approaches (Dhamala et al., 2008); conditional and pairwise approaches (Smith et al., 2011). More formally, typical approach considers a linear Gaussian vector auto-regressive (VAR) linear model between the variables, in this case, states of neurons *X*_*i*_ (Lütkepohl, 2005),


$$X_{i}\left(t\right)=\overset{N}{\underset{j=1}{\sum }}\overset{K}{\underset{k=1}{\sum }}A_{ji}\left(k\right)X_{j}\left(t-k\right)+\epsilon_{i}\left(t\right)$$

where *K* is the maximum number of lags (model order) and *A*_*ji*_(*K*) are real-valued linear regression coefficients, and $\epsilon_{i}\left(t\right)\sim N\left(0,\sigma^{2}I\right)$. Neuron *j* is said to *Granger-cause* neuron *i* if at least one of the coefficients *A*_*ji*_(*k*)≠0 for *k* = 1, …, *K*. In practice, *A*_*ji*_(*k*) are estimated by minimizing the squared prediction error or by maximizing the likelihood or sparsity-inducing penalized likelihood (Pollonini et al., 2010; Basu et al., 2015). Granger Causality has been applied toward inference of CFC in the linear Gaussian VAR model setting (Pollonini et al., 2010; Schmidt et al., 2016; Guo et al., 2020). Extensions of GC to categorical random variables, non-linear auto-regressive models and non-parametric models exist (Dhamala et al., 2008; Marinazzo et al., 2008; Tank et al., 2017, 2018).

GC was first introduced within econometrics and later has been used to find directed functional connectivity in electrophysiological studies (Granger, 1969; Geweke, 1984), in EEG or MEG datasets, either at source or sensor level (Bernasconi and KoÈnig, 1999; Ding et al., 2000; Brovelli et al., 2004; Barrett et al., 2012). The slow dynamics and regional variability of the haemodynamic response to underlying neuronal activity in fMRI were shown to be able to confound the temporal precedence assumptions of GC (Roebroeck et al., 2005; Bressler et al., 2008; David et al., 2008; Wen et al., 2012).

While Granger Causality provides a powerful tool for understanding which neural time series have a key role in predicting the future of other neural time series (Dahlhaus and Eichler, 2003; Stokes and Purdon, 2017; Guo et al., 2018), studies express concern since prediction is not a formal setting to answer causal questions related to the consequence of interventions and counterfactuals (Friston, 2009; Eichler, 2013; Grassmann, 2020). Furthermore, in practice, GC uses a model assumption between the variables, e.g., a linear Gaussian VAR model, and results could differ when this assumption does not hold (Lütkepohl, 2005). Notwithstanding the limitations, GC has been a well-known method in the neural time series scenarios, and applications (Qiao et al., 2017; Guo et al., 2020). GC based on linear VAR model is equivalent to Transfer Entropy for Gaussian variables, while the latter is a non-linear method in its general formulation (Barnett et al., 2009). Transfer Entropy has been explored as a tool to explore connectomics at different scales (Ursino et al., 2020), and also applied to the retina circuit (Wibral et al., 2013).

### 3.3. Dynamic Causal Model

**Figure 4:**
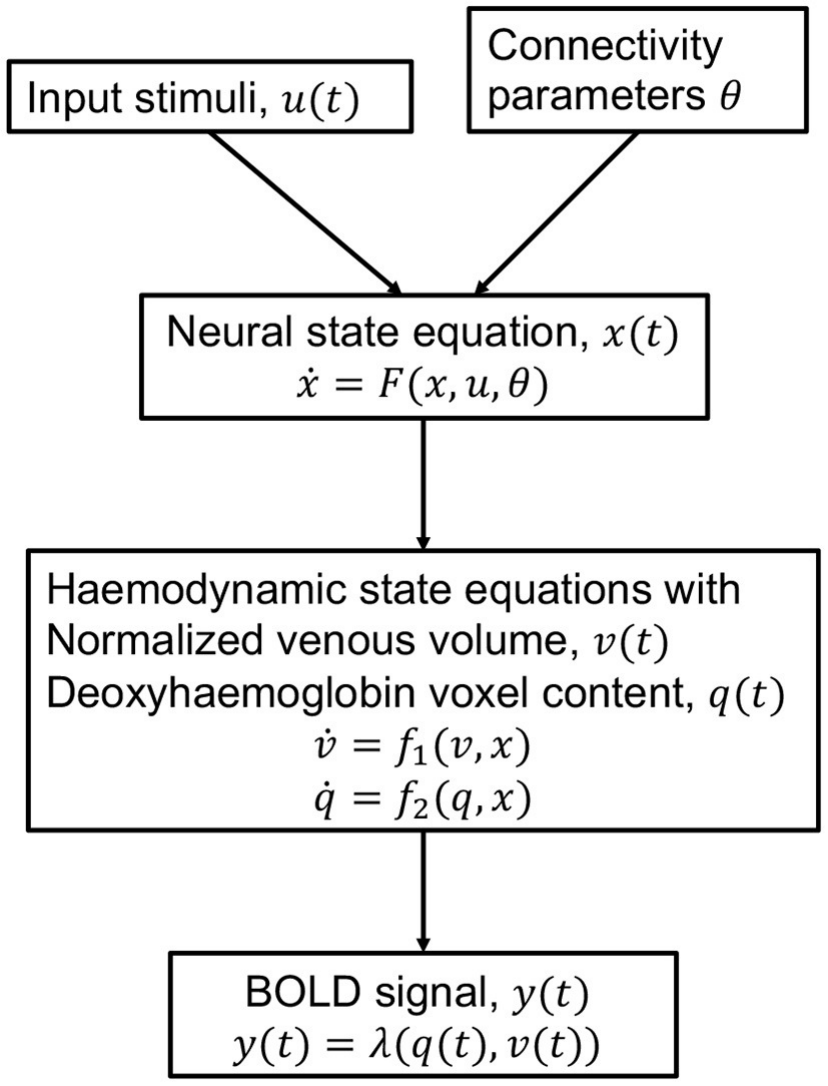
Schematic of the haemodynamic model used by DCM for fMRI. Input stimuli *u*(*t*) lead to neural state *x*(*t*) subject to connectivity parameters θ modeled by the neural state equation. Neuronal activity leads to increase in blood flow that changes in venous volume *v*(*t*) and deoxyhaemoglobin content *q*(*t*) modeled by the haemodynamic state equations. These haemodynamic states are fed to a forward non-linear model λ which results in the Blood Oxygenation Level Dependent (BOLD) fMRI response (Stephan et al., 2007b).

The Dynamic Causal Model (DCM) is an approach for modeling brain activity and causal interaction between brain regions (See Figure 4). DCM was first introduced for fMRI time series and treats the brain as a deterministic non-linear dynamical system network in which neural activity is considered to be unobserved and propagates in an input-state-output system (Friston et al., 2003). The system is attached to a forward model that maps the neural activity to observed Blood Oxygenation Level Dependent (BOLD) fMRI signal (Friston et al., 2000; Stephan et al., 2007a,b). The neural and observational models are specified by a particular parametric form that depends on the data and imaging modality and they together form a fully generative model (Friston et al., 2013). DCM outputs evidence for different neural and/or observational models and posterior parameter estimates optimized by variational Bayesian techniques (Penny, 2012). The coupling parameters between hidden states for different brain regions, in the differential equations that specify the model, constitute the CFC. The DCM model incorporates interactions due to the experimental equipment and changes due to external perturbations (Marreiros et al., 2010).

DCM compares the evidence for different hypothesized models, and thereby is a tool for testing hypothesis for model and experimental design. DCMs are not restricted to linear systems and include intricate models with large number of parameters for neural dynamics and experimental perturbations. The model is typically constructed to be biologically plausible. Constraints are exercised to the model through priors. The priors are useful to specify which connections are believed to be more likely. A Bayesian likelihood with the prior distribution is maximized to obtain the parameter estimates. The approach relies on precise modeling and aims to specify a biologically plausible detailed mechanistic model of the neuronal dynamics and imaging equipment perturbation (Stephan et al., 2010). While DCM was originally formulated to be deterministic, recent advances can include stochastic fluctuations in the neural activity as well (Stephan et al., 2008; Li et al., 2011). The DCM framework has also been extended beyond fMRI and established in the magneto/encephalography domain (Kiebel et al., 2008), and in local field potentials (Moran et al., 2013).

### 3.4. Directed Probabilistic Graphical Models

*Directed Probabilistic Graphical Models* (DPGMs) provide a probabilistic foundation to causality in a manner that answers causal queries through a generalized model without requiring specific modeling of the neural dynamics. An important aspect to take into account in inference of CFC is stochasticity. Neural signals are stochastic due to noise and intrinsic nature of neuron firing (Manwani and Koch, 1999; Stein et al., 2005). The variability and noise in neural dynamics is well known to challenge the determination of neural phenomenon, e.g., the detection of onset of epileptic seizures (Biswas et al., 2014; Vidyaratne and Iftekharuddin, 2017). So, when we say “spike in activity of neuron A is a cause of the spike in activity of neuron B,” the cause makes the effect more likely and not certain due to other factors like incoming inhibitory projections from other neurons and extracellular ion concentration (Kroener et al., 2009; Soybaş et al., 2019). Moreover, additional arbitrary functional relationships between each cause and effect could exist such that these introduce arbitrary disturbances following some undisclosed probability distribution. For example, it is widely known that diet and stress in humans change the levels of neurotransmitters in the brain (Fernstrom, 1977; Mora et al., 2012). Thereby, the strength of causal relationships between neurons can be perturbed by daily variability in diet and/or stress. Also, uncertainties in effect can occur from unobserved causes which is especially true in the context of neural signals as extraneous variables such as diet and stress are often not observed or due to recording from only a fraction of the units in the brain (Krishnaswamy et al., 2017). With these considerations, it is elaborated that values of exogenous variables do not imply values for the remaining variables in a deterministic manner. This motivates the need to consider a probabilistic foundation to causation and causal graphs provided by DPGM. We outline three conditions of DPGM that connect probabilities with causal graphs: The Directed Markov Property, the Causal Minimality Condition, and the Faithfulness Condition.

**Figure 5:**
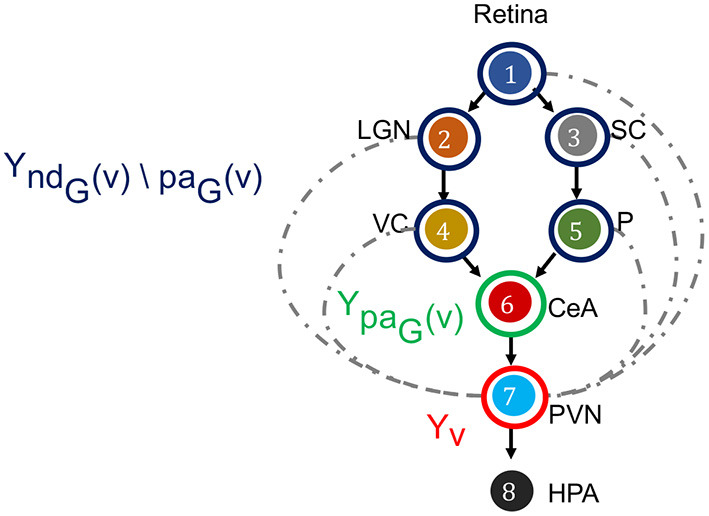
Directed Markov Property in the context of fear stimulus. The DAG in the example of Figure 3 is annotated to illustrate the Directed Markov Property (Equation 4). PVN is selected as node *v*, its random variable is hence *Y*_*v*_ (red). The parents of v denoted as *pa*_*G*_(*v*), and corresponding random variables are *Y*_*p*_*a*__*G*_(*v*)_ (green). The non-descendants of *v* before parents, denoted as *nd*_*G*_(*v*)\*pa*_*G*_(*v*), and corresponding random variables *Y*_*n*_*d*__*G*_(*v*)\*pa*_*G*_(*v*)_ (blue). Directed Markov Property holds with the true causal edges (black), as for them parents and children are functionally related (Equation 6). Causal Minimality Condition ensures that potential edges (blue dotted) between *nd*_*G*_(*v*)\*pa*_*G*_(*v*) nodes and *v* are absent from the DAG.

Let *G* = (*V, E*) be a DAG over neuron labels *V* = (*v*_1_, …, *v*_*N*_) with directed edges *E* (e.g., Figure 3). DPGM typically considers the graph to be a DAG because of coherent probability semantics with a DAG and challenges with directed cycles, while there could be more general extensions (Lauritzen, 2001; Maathuis et al., 2019). Nodes *v* and *u* ∈ *V* are said to be *adjacent* if *v*→*u* ∈ *E* or *u*→*v* ∈ *E*. A *path* is a sequence of distinct nodes in which successive nodes are adjacent. If π = (*v*_0_, …, *v*_*k*_) is a path then *v*_0_ and *v*_*k*_ are the end-points of the path. If every edge of π is of the form *v*_*i*−1_→*v*_*i*_ then *v*_0_ is an *ancestor* of *v*_*k*_ and *v*_*k*_ is a *descendant* of *v*_0_. We use the convention that *v* is an ancestor and descendant of itself. The set of *non-descendants* of *v*, denoted *nd*_*G*_(*v*), contains nodes *u* ∈ *V* that are not descendants of *v*. The set of *parents* of *v* ∈ *V* is denoted as *pa*_*G*_(*v*) = {*u* ∈ *V*:*u*→*v* ∈ *E*}. We mark the set *nd*_*G*_(*v*)\*pa*_*G*_(*v*) as the set that contains all nodes which are older ancestors of *v* before its parents (Figure 5). Let *Y*_*v*_ denote a scalar-valued random variable corresponding to *v* ∈ *V*, e.g., the neural recording at time *t*: *Y*_*v*_ = *X*_*v*_(*t*), average of recordings over time $Y_{v}=\bar{X}\left(v\right)$, and for a set of neurons *A*⊂*V*, *Y*_*A*_ denotes the random vector (*Y*_*v*_, *v* ∈ *A*). With these notations, we outline the three conditions of DPGM.

#### 3.4.1. Directed Markov Property

(*Y*_*v*_, *v* ∈ *V*) is said to satisfy the *Directed Markov Property* with respect to the DAG *G* if and only if,


(4) $$\begin{array}{r} Y_{v}⊥⊥Y_{nd_{G}\left(v\right)\backslash pa_{G}\left(v\right)}|Y_{pa_{G}\left(v\right)} \end{array}$$

for every *v* ∈ *V*. The Directed Markov Property translates the edges in the DAG into conditional independencies, such that each node *Y*_*v*_ and its older ancestors *Y*_*n*_*d*__*G*_(*v*)\*pa*_*G*_(*v*)_ are conditionally independent given its parents *Y*_*p*_*a*__*G*__(*v*). In other words, the influence of each node's ancestors beyond parents reaches to the node exclusively via its parents. In this way, the Directed Markov Property connects probabilistic conditional independencies with relationships of causal influence between nodes of a directed graph. For example, under the Directed Markov Property, in Figure 5, the assertion that the activity of VC and P are conditionally independent of the activity of PVN, given the activity of CeA at time *t* corresponds to the causal relationship that the influence of the activity of VC and P on the activity of PVN is mediated by the activity of CeA, represented in the DAG as CeA a parent node of PVN, and VC and P are non-descendant nodes beyond parents of CeA.

The Directed Markov Property for DPGM (DMP) is different from the Markov Property for undirected PGM (MP) in that DMP relates conditional independencies between random variables in a directed graph to causal relationships in the directed graph, whereas MP relates conditional independencies between random variables in an undirected graph to edges of association in the undirected graph. Yet, both incorporate multi-nodal interactions in the graphs beyond a pairwise manner. Furthermore, we had seen in Theorem 1 that MP yields a factorization of the probability density of the random variables comprising the PGM. The DMP also yields a factorization of the joint probability density for DPGM in an adapted manner as follows (Verma and Pearl, 1988).

** Theorem 2 (Factorization Theorem for DPGM)**. For (*Y*_*v*_:*v* ∈ *V*) real random variables with density *f* with respect to a product measure, it satisfies the Directed Markov Property (Equation 4) with respect to the DAG *G* if and only if their distribution factorizes according to the *G*, which means,


(5) $$\begin{array}{r} f\left(y\right)=\underset{v\in V}{\prod }f\left(y_{v}|y_{pa_{G}\left(v\right)}\right),y\in {\mathbb{R}}^{V} \end{array}$$

where *f* is the density of *Y*_*v*_, and *f*(*y*_*v*_|*y*_*p*_*a*__*G*_(*v*)_) are conditional probability densities.

The Directed Markov Property can be equivalently represented with functional relationships between parent and child instead of conditional independencies, which is described in the following theorem (Bollen, 1989).

** Theorem 3 (Functional Equivalence of DPGM)**. If *Y*_*v*_ satisfies


(6) $$\begin{array}{r} Y_{v}=g_{v}\left(Y_{pa_{\tilde{G}}\left(v\right)},\epsilon_{v}\right),v\in V \end{array}$$

where ϵ_*v*_ are independent random variables and *g*_*v*_ are measurable functions for *v*∈*V* and $\tilde{G}$ is a DAG with vertices *V*, then *Y*_*v*_, *v* ∈ *V* satisfies the Directed Markov Property with respect to $\tilde{G}$. Conversely, if *Y*_*v*_, *v* ∈ *V* satisfies the Directed Markov Property with respect to a DAG $\tilde{G}$, then there are independent random variables ϵ_*v*_ and measurable functions *g*_*v*_ for which (Equation 6) holds.

Since the functional equations admit a natural causal interpretation, so do DPGMs satisfying the Directed Markov Property (Drton and Maathuis, 2017). The variables in $Y_{pa_{\tilde{G}}}\left(v\right)$ are direct causes of *Y*_*v*_, meaning that changes in $Y_{pa_{\tilde{G}}}\left(v\right)$ lead to changes in distribution *Y*_*v*_, but not necessarily the other way around. Furthermore, when *Y*_*v*_, *v* ∈ *V* satisfies the Directed Markov Property, then Equation (6) holds for some choice of functions {*g*_*v*_} and error distributions {ϵ_*v*_}, which implies causal relationships among *Y*_*v*_, *v* ∈ *V*.

DPGMs can predict the consequence of a counterfactual intervention on the random variables (Pearl, 2009a). Using Theorem 3 we show in the following that we only need to remove the edges pointing to the intervened random variables in the DPGM to incorporate the impact of a counterfactual intervention. More precisely, if before the intervention, *G* is DPGM and *Y*_*v*_, *v* ∈ *V* satisfies the DMP with respect to *G*, an intervention to the random variables will modify (Equation 6) only those variables that are impacted by the intervention. For example, let us consider the intervention forcing *Y*_*v*_0__ to take the value 0 or 1 regardless of value at other nodes. This intervention will change (Equation 6) by excluding the equations in which *Y*_*v*_0__ is a function of other nodes. This corresponds to replacing *pa*_*G*_(*v*_0_) by an empty set in (Equation 6), and in other words, removing the edges pointing to node *v*_0_ in *G*. That is, after the intervention, (Equation 6) holds with a different graph *G*′ that is obtained by removing the edges incident upon the intervened nodes in *G*. Equivalently, by Theorem 3, after the intervention *Y*_*v*_, *v* ∈ *V* satisfies DMP with respect to *G*′, and thus *G*′ is the DPGM after the intervention.

#### 3.4.2. Causal Minimality Condition

Let (*Y*_*v*_, *v* ∈ *V*) satisfy the DMP with respect to the DAG *G*. *G* satisfies the *Causal Minimality Condition* if and only if for every proper subgraph *H* of *G* with vertex set *V*, (*Y*_*v*_, *v* ∈ *V*) does not satisfy the DMP with respect to *H*. In other words, if adding any edge on to *G* can also satisfy the DMP, we do not add such an edge, and consider the minimal graph *G* with respect to which DMP is satisfied to be the DPGM of *Y*_*v*_, *v* ∈ *V*. In principle, since the complete set of causes is unknown, there can be multiple graphs that would fit a given distribution of random variables for DMP, each connecting the observed variables through different causal relationships. Among all those possible graphs satisfying DMP, the causal minimality condition considers the simplest one and ensures a unique causal DPGM.

#### 3.4.3. Faithfulness Condition

The Directed Markov Property with respect to a DAG *G* prescribes a set of conditional independence relations on the random variables comprising the graph. However, in general, a probability distribution *P* of random variables in DAG *G* that has the independence relations given by the DMP may also include other independence relations. If that does not occur such that all the conditional independence relations by the probability distribution *P* are encompassed by *G*, then we denote *P* and *G* as *faithful* to one another.

#### 3.4.4. Inference of DPGM

Several methods have been developed for inferring DPGM with the Directed Markov Property, Causal Minimality and Faithfulness conditions for stationary observed data. These include the PC algorithm (constraint-based, acyclic graph, no latent confounders, no selection bias) (Spirtes et al., 2000), FCI algorithm (constraint-based, acyclic graph, latent confounders, selection bias) (Spirtes et al., 1999), GES (score-based equivalence class search) (Chickering, 2002) and GIES (score-based equivalence class search from data with multiple interventions) (Hauser and Bühlmann, 2012).

**Figure 6:**
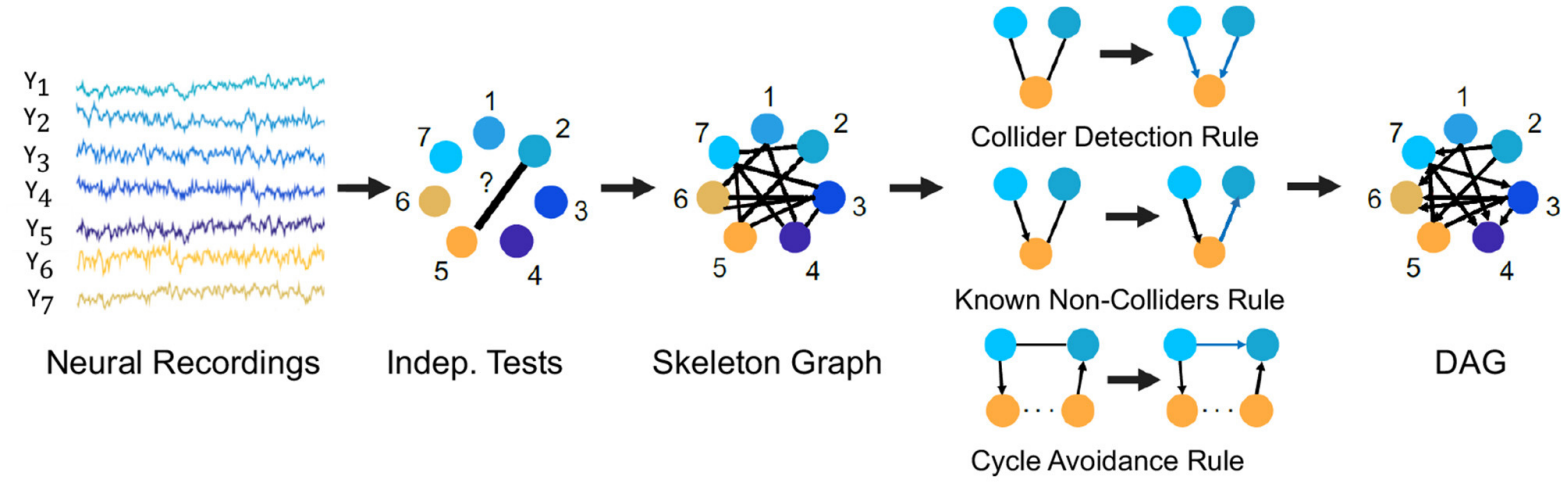
The PC algorithm. Steps of the PC algorithm to infer the DPGM from observed data are summarized by five diagrams (left to right). Data for variables *Y*_1_−*Y*_7_ is visualized in the context of neural recordings. Graph with nodes 1−7 corresponding to variables *Y*_1_−*Y*_7_ has no edges. Then, an edge introduced between *Y*_*i*_ and *Y*_*j*_ if they are independent or conditionally independent given any other variable(s) determined by statistical tests, which results in the undirected Skeleton Graph. Using rules of converting undirected to directed edges as depicted in the figure-Collider Detection rule, Known Non-Colliders rule and Cycle Avoidance rule, the skeleton graph is converted to a DAG.

For example, we describe here the PC algorithm which is a popular statistical tool to infer causal connections between stationary random variables under independent and identically distributed sampling. It is a constraint-based method in which a consistent statistical test for conditional independence is used to select the connectivity graph among the random variables of interest. For Gaussian random variables and linear relationships, a standard choice for such a conditional independence test is constructed using the Fisher's Z-transform (Kalisch and Bühlmann, 2007). For non-Gaussian variables and non-linear relationships, kernel and distance based tests of conditional independence are used (for e.g., the Kernel PC algorithm Tillman et al., 2009). The algorithm first represents the observed variables by nodes of a graph and starts with an empty set of edges and decides whether to put an undirected edge between each pair of nodes, e.g., node 2 and 5 in Figure 6. In order to determine whether to have an edge between the pair of nodes, it performs consistent statistical tests for independence of the random variables for the pair of nodes or conditional independence given the random variables for other node(s). If any of the tests finds evidence for independence or conditional independence, an edge is drawn between the pair of nodes and otherwise no edge is drawn between the pair of nodes. The process is followed for each pair of nodes to result in an undirected graph, called the skeleton graph. Using rules such as the Collider Detection rule, Known Non-Colliders rule and Cycle Avoidance rule, as depicted in Figure 6, the undirected edges are directed to convert the skeleton graph into a DAG. Figure 6 provides a schematic of the algorithm with the context of neural recordings. The PC algorithm is shown to be consistent for finding the true causal graph in the absence of latent confounders (Spirtes et al., 2000).

### 3.5. Comparitive Study of Approaches to Causal Functional Connectivity

**Figure 7:**
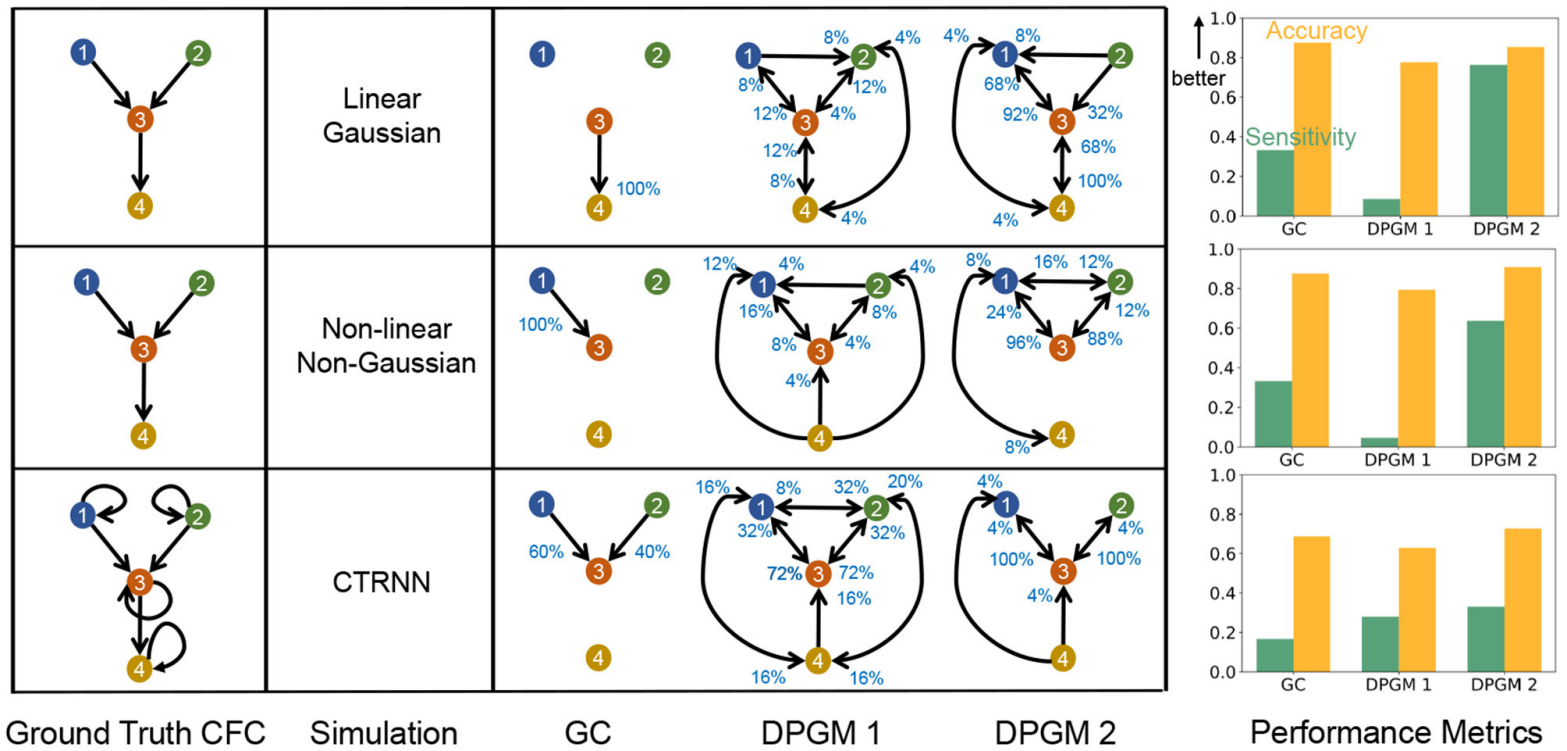
Comparative study of CFC inference. CFC inference of GC, DPGM 1 and DPGM 2 methods is compared on three examples of motifs and simulation paradigms; from top to bottom: Linear Gaussian, Non-linear Non-Gaussian, CTRNN. Table: 4 neurons motifs that define the Ground Truth CFC (left) are depicted side by side with inferred CFC over several simulation instances according to the three different methods (right). An edge *v*→*w* in each inferred CFC corresponds to an edge detected in any of the inference instances. The percentage (blue) next to each edge indicates the number of times out of all instances that the edge was detected. Right: For each motif and simulation paradigm, Sensitivity (green) and Accuracy (orange) of each method is shown.

We compare the performance of exemplary approaches of CFC inference discussed above to recover relationships in ground truth dynamical equations by generating synthetic data from three simulation paradigms and estimate their CFC using the methods of GC and DPGM (see Figure 7). The simulation paradigms correspond to specific model assumptions to assess the impact of model assumptions on the performance of the approaches.

1. Linear Gaussian Time Series (Figure 7 top-row). Let *N*(0, 1) denote a standard Normal random variable. We define *X*_*v*_(*t*) as a linear Gaussian time series for *v* = 1, …, 4 whose true CFC has the edges 1 → 3, 2 → 3, 3 → 4. Let *X*_*v*_(0) = *N*(0, 1) for *v* = 1, …, 4 and for *t* = 1, 2, …, 10000,
$$\begin{array}{l} X_{1}\left(t\right)=1+N\left(0,1\right),\\ X_{2}\left(t\right)=-1+N\left(0,1\right),\\ X_{3}\left(t+1\right)=2X_{1}\left(t\right)+X_{2}\left(t\right)+N\left(0,1\right),\\ X_{4}\left(t+1\right)=2X_{3}\left(t\right)+N\left(0,1\right) \end{array}$$
2. Non-linear Non-Gaussian Time Series (Figure 7 middle-row). Let *U*(0, 1) denote a *Uniformly* distributed random variable on the interval (0, 1). We define *X*_*v*_(*t*) as a non-linear non-Gaussian time series for *v* = 1, …, 4 whose true CFC has the edges 1 → 3, 2 → 3, 3 → 4. Let *X*_*v*_(0) = *U*(0, 1) for *v* = 1, …, 4 and for *t* = 1, 2, …, 10000,
$$\begin{array}{l} X_{1}\left(t\right)=U\left(0,1\right),\\ X_{2}\left(t\right)=U\left(0,1\right),\\ X_{3}\left(t+1\right)=4\sin\left(X_{1}\left(t\right)\right)+3\cos\left(X_{2}\left(t\right)\right)+U\left(0,1\right),\\ X_{4}\left(t+1\right)=2\sin\left(X_{3}\left(t\right)\right)+U\left(0,1\right) \end{array}$$
3. Continuous Time Recurrent Neural Network (CTRNN) (Figure 7 bottom-row). We simulate neural dynamics by Continuous Time Recurrent Neural Networks, Equation (7). *u*_*j*_(*t*) is the instantaneous firing rate at time *t* for a post-synaptic neuron *j*, *w*_*ij*_ is the linear coefficient to pre-synaptic neuron *i*'s input on the post-synaptic neuron *j*, *I*_*j*_(*t*) is the input current on neuron *j* at time *t*, τ_*j*_ is the time constant of the post-synaptic neuron *j*, with *i, j* being indices for neurons with *m* being the total number of neurons. Such a model is typically used to simulate neurons as firing rate units
(7) $$\begin{array}{r} \tau_{j}\frac{du_{j}\left(t\right)}{dt}=-u_{j}\left(t\right)+\overset{m}{\underset{i=1}{\sum }}w_{ij}\sigma \left(u_{i}\left(t\right)\right)+I_{j}\left(t\right),j=1,\ldots ,m. \end{array}$$
  We consider a motif consisting of 4 neurons with *w*_13_ = *w*_23_ = *w*_34_ = 10 and *w*_*ij*_ = 0 otherwise. We also note that in Equation (7), activity of each neuron *u*_*j*_(*t*) depends on its own past. Therefore, the true CFC has the edges 1 → 3, 2 → 3, 3 → 4, 1 → 1, 2 → 2, 3 → 3, 4 → 4. The time constant τ_*i*_ is set to 10 msecs for each neuron *i*. We consider *I*_*i*_(*t*) to be distributed as independent Gaussian process with the mean of 1 and the standard deviation of 1. The signals are sampled at a time gap of *e*≈2.72 msecs for a total duration of 10 secs.

For these network motifs we compare the methods GC, DPGM 1 and DPGM 2. We compute the GC graph using the *Nitime* Python library which fits an MVAR model followed by computation of the Granger Causality by the *GrangerAnalyzer* (Rokem et al., 2009).

We compute DPGM using the PC algorithm which requires several samples of a scalar-valued random variable *Y*_*v*_ (measured activity) for neurons *v* ∈ *V*. We consider two of such *Y*_*v*_ possibilities

DPGM 1: Neural recordings at time *t*: *Y*_*v*_ = *X*_*v*_(*t*), *v* ∈ *V*. Different *t* gives different samples of *Y*_*v*_.
DPGM 2: Windowed Average of recordings over a duration of 50 msec: $Y_{v}={\bar{X}}_{v},v\in V$, and averaging over different windows of 50 ms with a gap of 50 ms in between consecutive windows gives different samples of *Y*_*v*_.

We quantified the performance of the algorithms by inference of CFC for 25 different simulations and summarize the performance by two metrics, Accuracy (A) and Sensitivity(S). Let True Positive (TP) be the number of correctly identified edges, True Negative (TN) be the number of missing edges that were correctly identified, False Positive (FP) be the number of incorrectly identified edges and False Negative (FN) be the number of missing edges incorrectly identified across simulations. We define the Accuracy as


$$A=\frac{\text{TP+TN}}{\text{TP+TN+FP+FN}},$$

which measures the ratio of the count of correctly identified edges or missing edges to the count of all possible edges across simulations. In the motifs and simulation paradigms we consider, there are 4 neurons and 16 possible edges (including self-loops) per simulation resulting with total of 400 possible edges across 25 simulations. We also define the Sensitivity as


$$S=\frac{\text{TP}}{\text{TP}+\text{FN}}$$

the ratio of the count of true edges that were correctly identified to the total count of the true edges across simulations. In this comparative study, Sensitivity is more relevant than Accuracy since it focuses on the detection of the true edges. Indeed, in the extreme case of having the estimated CFC to be an empty set of edges across simulations, the linear Gaussian paradigm will still have 70% neuron pairs correctly identified to be not connected by an edge, thereby resulting in *A* = 70%. Whereas, there will be 0% of true edges detected correctly resulting in *S* = 0%, which reflects the undesirability of the empty graph estimate. We report both Accuracy and Sensitivity for a comprehensive summary of performance. We also report the percentage of the simulations that has each estimated edge present. Higher percentage indicates higher confidence in the detection of that edge. Figure 7 compares the results for GC, DPGM 1 and DPGM 2 methods in inference of the true CFC.

In *Linear Gaussian scenario (top row in* *Figure 7**)*, the connections between neurons in the Ground Truth CFC are excitatory due to positive coefficients in the linear dynamical equation for neural activity. GC generates a sparse set of edges in which it correctly detects a single edge 3 → 4 among the three edges of the true CFC. DPGM 1 generates a large set of edges (9 out of 16 possible) with several of them being spurious. Indeed, each edge is present in less than 25% of the simulations. DPGM 2 generates the same number of edges as DPGM 1, however it has less spurious edges indicated by higher percentages for the expected edges in the Ground Truth CFC (1 → 3, 3 → 4 with 92% and 100%). Overall, all methods result in *A*>80% while sensitivity for GC, DPGM 1 and DPGM 2 varied significantly *S* = 33.3%, 8.7%, 76.3%, respectively. We thereby conclude that among the three methods, GC is the most accurate but since it did not detect two out of three edges, it is not as sensitive as DPGM 2.

In *Non-linear Non-Gaussian scenario (middle row)*, in the Ground Truth CFC for the Non-linear Non-Gaussian scenario, 1 → 3, 3 → 4 are excitatory due to sin(*x*) being an increasing function while 2 → 3 is an inhibitory connection due to cos(*x*) being a decreasing function for *x* ∈ [0, 1] in their dynamical equation. As previously, GC consistently detects a sparse set of edges (single edge 1 → 3 with 100%) which is one of the three true edges. DPGM 1 obtains a large set of edges with several of them are spurious edges and all edges appear in less than 25% of the trials. For DPGM 2, the number of spurious edges obtained is more than GC and less than DPGM 1. DPGM 2 obtains correctly two out of the three true edges 1 → 3 and 2 → 3 in most of the trials (96 and 88%, respectively). In summary, GC, DPGM 1 and DPGM 2 resulted in an accuracy of *A* = 87.5%, 79.4%, 90.8%, respectively and sensitivity of *S* = 33.3%, 4.7%, 63.7%, respectively. For this scenario, DPGM 2 has the highest accuracy and sensitivity among the methods.

In *CTRNN scenario (bottom row)*, self-loops are present for each neuron, and due to positive weights and increasing activation function σ(·) in their dynamical equation, the connections in the Ground Truth CFC are excitatory. GC obtains two of the three true non-self edges 1 → 3, 2 → 3 for 60%, 40% of the trials. DPGM 1 detects spurious edges, but also infers the true edges 1 → 3, 2 → 3 for 72% of the trials. DPGM 2 obtains less number of spurious edges compared to DPGM 1 and obtains all of the non-self true edges 1 → 2 and 2 → 3 for 100% of the trials. In summary, all methods result in a lower accuracy of *A*≈70% compared to other scenarios since they do not include self-loops and sensitivity is *S* = 16.7%, 28%, 33.2% for GC, DPGM 1 and DPGM 2, respectively, which is considerably lower than other scenarios. Among all methods, DPGM 2 obtains the highest accuracy followed by GC and lastly DPGM 1. DPGM 2 had the highest sensitivity compared to the other methods.

The choice of thresholds tunes the decision whether a connection exists in the CFC. For DPGM-based approaches, increasing the *p*-value cut-off for conditional independence tests increases the rate of detecting edges while also increasing the rate of detecting false positives. We use a *p*-value cut-off of 0.1, after trial and error, for DPGM 1 and DPGM 2. For GC, a likelihood ratio statistic *L*_*uv*_ is obtained for testing *A*_*uv*_(*k*) = 0 for *k* = 1, …, *K*. An edge *u*→*v* is outputted if *L*_*uv*_ has a value greater than a threshold. We use a percentile-based threshold (Schmidt et al., 2016), and output an edge *u*→*v* if *L*_*uv*_ is greater than 95 percentile of *L*_*ij*_'s over all pairs of neurons (*i, j*) in the graph. We also trialed with different percentile thresholds of 90 percentile and higher, none of which outperformed DPGM.

## 4. Discussion

In this paper, we establish a statistical guide to neural connectomics with regards to mapping neural interaction anatomy, function and causality with the means of graphical models. We first describe possibilities of mapping neural anatomical connections with a graph, i.e., anatomical connectome, and demonstrate the difference between such a mapping and a graph that captures functional interactions, i.e., associative functional connectome (AFC). Recognizing that the ultimate goal of functional connectomics is to infer causal interactions between neurons, we define the graphical tools and properties needed to distill AFC into a directional graph, i.e., causal functional connectome (CFC), which represents flows of cause and effect in the interaction between neurons. We then compare exemplary common approaches having the ultimate goal of finding "causation," such as Granger Causality (GC), Dynamic Causal Model (DCM) and Directed Probabilistic Graphical Model (DPGM), in the context of functional connectomics. In particular, we introduce the developments in statistical theory of DPGM to the subject of CFC inference, and define the Directed Markov Property that guarantees consideration of cause and effect in graph settings. We show that this property is key in the definition of probabilistic graphical models that could constitute neural CFC. We then describe the PC algorithm, a common statistical approach for inference of such graphs. Based on these notions and the outcomes of the Directed Markov Property we formulate criteria based on which CFC models can be compared.

We conclude by performing a holistic comparison, in Table 1, of several common approaches that do not obey the Directed Markov Property, such as Granger Causality (GC) and Dynamic Causal Model (DCM), with variants of the PC algorithm (DPGM), comparing them with respect to the criteria that we have outlined. We demonstrate the applicability and the challenges for inference of CFC from measured neural activity for each of the approaches on simulated motifs in Figure 7.

**Table 1:** Comparative summary of different approaches for causal modeling in functional connectomics.

	**GC**	**DCM**	**DPGM**
Format of Causality	Non-zero parameters in VAR model	Coupling parameters in biological model	Directed Markov Graph
Inclusion of temporal relationships	** Yes **	** Yes **	**No**, formulation for stationary variables
Generalizable statistical model	** Yes **	** No **	** Yes **
Non-parametric Model	**Yes**, parametric and non-parametric approaches exist.	**No**, biologically mechanistic non-linear model.	**Yes**, equivalent to an arbitrary functional relationship between nodes.
Supports CFC estimation	** Yes **	**No**, suitable for comparing model hypotheses	** Yes **
Occurrence of cycles (including self-loops) in the model	**Yes** (neuron *i*→*i* when *A*_*ii*_(*k*)≠0 for some *k*)	**Yes** (*i*→*i* when θ_*ii*_≠0)	**No**, it is a DAG
Incorporation of interventional and counterfactual queries	** No **	** No **	**Yes** but for stationary variables.

Functional Connectivity between neurons describes statistical dependency between observed neural signals and is descriptive in nature without requiring accurate modeling. FC can either describe undirected statistical associations (AFC) or directed causal interdependencies among the observed neural signals (CFC) (Bastos and Schoffelen, 2016). In contrast, there is another concept of Effective Connectivity in literature which aims to quantify the causal influence between hidden neural states and corresponds to the parameter of a mechanistic model that aims to explain the observed directed dependencies (Stephan and Friston, 2009; Friston, 2011). In practice, the analysis of Effective Connectivity involves comparison of generative models with coupling among hidden brain states, based on evidence from observed data. Whereas, analysis of CFC is predictive in nature, that estimates the presence and/or strength of causal dependencies from the observed recordings and does not require generative modeling.

In this work, our aim is to formulate statistical properties and criteria related to causality of functional connectomics, rather than propose a new approach for causal functional connectome modeling. Such formulation is expected to identify existing gaps in causal modeling and guide extensions of causal functional connectome models ideally satisfying all criteria that we have outlined. Indeed, capturing as many causal criteria is fundamental to any approach from statistical and application points of view. For example, one such property of importance is the ability to uncover directed relationships in ground truth dynamical equations (Schmidt et al., 2016; Reid et al., 2019), which we include in our comparative study. We have compared the approaches to find CFC in simulations from linear gaussian, non-linear non-gaussian, and CTRNN to demonstrate how specific model assumptions in ground truth dynamical equations are impacting the utilization of the approaches in recovery of relationships between the neurons. For the methods that we have tested, our simulated comparison shows that GC output typically results in a sparse graph with inferred edges that indeed represent causal connections, but we find that it also misses multiple edges that represent causal connections (high accuracy; low sensitivity). For DPGM, we find that the output depends on the choice of measured activity. DPGM 1, which uses full neural time series, results in comparatively low accuracy and low sensitivity since does not capture dependencies across time. DPGM 2 uses neural activity averaged over time and results in comparatively high accuracy as well as sensitivity for detection of most of the true edges, but not all of them. These results are not surprising, since the PC algorithm guarantees causality for independent samples per time point thus guarantees the Directed Markov Property only for a single time point, however here we aim to infer a single CFC for the whole recording over time (as in DPGM 1) (Pearl, 2009a). In such a case, the Directed Markov Property and causality are not guaranteed. Averaging over time and separating by time gaps to reduce interdependence between samples leads to improved performance of DPGM 2, but this does not necessarily reflect the time dependent causes and effects between and within neural time series, thus again does not guarantee causality.

Our exposition of properties that each approach is based upon and the comparative study show that each of the methods address different aspects of modeling causality of neural interaction and mapping them in the form of a graph. In particular, GC aims to obtain the directed functional connectivity from observed neural states, in a way that tells whether a variable's *past* is predictive of another's future, without requiring a detailed model. It consists of a framework based on auto-regression models which is relatively easy to compute. In contrast, DCM enables *specific* model hypotheses to be compared based on evidence from data and provides insights on causal connectivity between hidden neural states based on those models. DPGM is a *generic* procedure that represents causal functional connectivity from observed neural states in a way that is predictive of the consequence of counterfactual interventions while answering queries of causal dependency in both interpretable and mathematically precise manner. To summarize these differences, we outline in Table 1 the strengths and weaknesses of each of the approaches with respect to applicability to various criteria of causality. Although these approaches are three distinct pathways popular for causal modeling, there have been attempts to combine them under a single framework (Eichler and Didelez, 2010), and to quantify causal effects that are applicable to either framework although under stringent conditions (Chicharro and Ledberg, 2012).

The comparative table demonstrates that with respect to the model that each approach is assuming, GC requires a linear model in its common use, though has recent non-linear and non-parametric extensions. DCM requires a strict well defined mechanistic biological model and thus can only compare different models based on evidence from data. In comparison, DPGM has an advantage that it does not require modeling of the neural dynamics using a parametric equation or assumption of a linear model. Furthermore, the Directed Markov Condition of the DPGM implies the existence of a functional relationship (Equation 6) between parent and children connections in the graph, thus doing away with the need for modeling by specific linear or non-linear functions. In regards to guarantee of causality, GC can provide useful insights into a system's dynamical interactions in different conditions, however its causal interpretation is not guaranteed as it focuses on the predictability of future based on past observations of variables. DCM uses the parameters for coupling between hidden neural states in competing biological models to indicate CFC, however it compares hypothetical models based on evidence from data which relevance to causality is not guaranteed (Friston et al., 2003). In comparison, DPGM provides a probabilistic foundation for causality which is predictive of the consequence of possible intervention like neuron ablation and counterfactual queries. Inference of CFC is possible with several causal graph inference algorithms such as the PC algorithm.

Such properties of causal interaction between entities are what makes DPGM popular in various disciplines such as genomics and econometrics (Friedman, 2004; Haigh and Bessler, 2004; Deng et al., 2005; Wang et al., 2005, 2017; Kalisch et al., 2010; Ebert-Uphoff and Deng, 2012; Mourad et al., 2012; Sinoquet and Mourad, 2014; Ahelegbey, 2016; Liu et al., 2018; Gómez et al., 2020). However, such an inference by DPGM typically produces a DAG, though adaptations exist which aim to include cycles in the CFC while having a more complicated output (Richardson and Spirtes, 1996). Furthermore, DPGM-based inference guarantees causality for independent measurements only. There is no such guarantee when considering whole neural time series due to temporal dependence, and this in practice leads to a decline in performance due to the degree of temporal dependence in the time series. Despite these limitations, DPGM substantially adds to the causal interpretation of CFC by being predictive of counterfactual interventions, unlike GC and DCM. DPGM is model-free, unlike popular GC variations relying on auto-regression and DCM. DPGM estimates the CFC graph from observed data, unlike DCM. Furthermore, using average activity over time instead of the entire time series, DPGM 2 performs well at the recovery of ground truth connectivity in simulation studies over different motifs compared to GC (see Figure 7).

In the context of fMRI datasets, several studies aim to evaluate the approaches. In simulated fMRI data from a DCM-based forward model, GC and its variations have suffered in performance (Ramsey et al., 2010; Smith et al., 2011), suspected to be due to inter-regional differences in the haemodynamic response function and causal processes occuring faster than the sampling rate. Later studies on more detailed fMRI simulations based on spiking neuron models coupled to biophysically realistic haemodynamic observation models revealed that, GC is largely invariant to changes in haemodynamic response function properties, however, in the presence of severe downsampling and/or high measurement noise, which can be typical of fMRI data, GC suffers in performance (Seth et al., 2013; Zhou et al., 2014; Solo, 2016). In an attempt to address these challenges, novel approaches to GC have recently been proposed (Winkler et al., 2016; Barnett and Seth, 2017; Faes et al., 2017). DCM exhibits good performance in comparing few model hypotheses for coupling between hidden neural states, however, DCM has the limitation that it is in general not mathematically and computationally feasible to search across all possible functional connectivity graphs and estimate from observed fMRI data (Friston et al., 2003). In contrast, DPGM-based approaches have been studied to perform relatively well in searching for the CFC and estimate from observed data in the fMRI setting (Ramsey et al., 2010; Mumford and Ramsey, 2014), such as, PC algorithm used on the whole time series (Smith et al., 2011), Independent Multisample Greedy Equivalence Search (IMaGES) which addresses Simpson's paradox in multi-subject fMRI data by assigning a penalized score for the CFC for each subject, combining them across individual subjects, and optimizing the score (Ramsey et al., 2010), and Fast Adjacency Skewness (FASK) which incorporates feedforward and feedback connections in the CFC graph in fMRI setting (Sanchez-Romero et al., 2019).

In conclusion, DPGM provides a probabilistic and interpretable formulation for CFC. We have established the statistical properties that the inferred DPGM should posses as well as demonstrated its performance in inference of CFC. While DPGM is a powerful causal framework, existing DPGM algorithms do not reflect the inter-temporal causal dependencies within and between the neural time series. Yet in the neural time series setting, nodes of the connectivity graph are neurons that correspond to an entire time series of neural activity and comprise inter-temporal dependence. Thus, the remaining challenge is the adaptation of the DPGM based formulation of CFC to incorporate inter-temporal dependencies in the neural time series. Such an adaptation will further increase the strength of using DPGM for CFC inference from neural dynamics.

## Data Availability Statement

The original contributions presented in the study are included in the article/supplementary material, further inquiries can be directed to the corresponding author.

## Author Contributions

RB and ES initiated the study and developed the methods, verified the results, and wrote and edited the manuscript. RB implemented the methods and performed comparative studies. Both authors contributed to the article and approved the submitted version.

## Funding

This work was supported in part by National Science Foundation grant IIS-2113003 and Washington Research Fund to ES.

## Conflict of Interest

The authors declare that the research was conducted in the absence of any commercial or financial relationships that could be construed as a potential conflict of interest.

## Publisher's Note

All claims expressed in this article are solely those of the authors and do not necessarily represent those of their affiliated organizations, or those of the publisher, the editors and the reviewers. Any product that may be evaluated in this article, or claim that may be made by its manufacturer, is not guaranteed or endorsed by the publisher.
